# Endoplasmic Reticulum Stress Promotes iNOS/NO and Influences Inflammation in the Development of Doxorubicin-Induced Cardiomyopathy

**DOI:** 10.3390/antiox10121897

**Published:** 2021-11-26

**Authors:** Ashim K. Bagchi, Akshi Malik, Gauri Akolkar, Davinder S. Jassal, Pawan K. Singal

**Affiliations:** 1 St. Boniface Hospital Albrechtsen Research Centre, Department of Physiology and Pathophysiology, Institute of Cardiovascular Sciences, University of Manitoba, Winnipeg, MB R2H 2A6, Canada; abagchi@sbrc.ca (A.K.B.); amalik@sbrc.ca (A.M.); dsjassal@sbgh.mb.ca (D.S.J.); 2 Cardio-Renal Division, Therapeutic Products Directorate, Ottawa, ON K1A 0K9, Canada; gauriakolkar@hotmail.com; 3 Section of Cardiology, Department of Internal Medicine, Rady Faculty of Health Sciences, University of Manitoba, Winnipeg, MB R2H 2A6, Canada

**Keywords:** Dox-induced cardiomyopathy, ER stress, inducible nitric oxide synthase, Toll-like receptor 2, apoptosis

## Abstract

Doxorubicin (Dox) is known to cause heart failure in some cancer patients. Despite extensive studies over the past half century, the subcellular basis of Dox-induced cardiomyopathy (DIC) is still elusive. Earlier, we suggested that Dox causes a delayed activation of unfolded protein response (UPR) which may promote mitochondrial Bax activity leading to cardiomyocyte death. As a follow up, using NO donor, S-Nitroso-N-acetyl-d,l-penicillamine (SNAP), and/or NOS inhibitor, N(ω)-nitro-L-arginine methyl ester (L-NAME), we now show that endoplasmic reticulum (ER) stress promotes inflammation through iNOS/NO-induced TLR2 activation. In vivo Dox treatment increased mitochondrial iNOS to promote ER stress as there was an increase in Bip (Grp78) response, proapoptotic CHOP (DDIT3) and ER-mediated Caspase 12 activation. Increased iNOS activity is associated with an increase in TLR2 and TNF-α receptor associated factor 2 (TRAF2). These two together with NF-κB p105/50 expression and a synergistic support through ER stress, promote inflammatory response in the myocardium leading to cell death and ultimately fostering DIC conditions. In the presence of NOS inhibitor, such detrimental effects of Dox were inhibited, suggesting iNOS/NO as key mediators of Dox-induced inflammatory as well as apoptotic responses.

## 1. Introduction

Irreversible and life-threatening cardiotoxicity is a serious side effect following anticancer therapy with doxorubicin (Dox) [1,2]. Dox-induced cardiotoxicity is very well characterized, whereby activated oxygen and other reactive oxygen species (ROS) as well as reactive nitrogen species (RNS) are reported to be involved [2,3,4,5,6,7,8,9,10]. ROS as well as RNS are now considered as key players in the inhibition of antioxidant enzymes and trigger for the initiation of various cell death pathways such as apoptosis and necrosis [8,11,12,13]. Studies on isolated cardiomyocytes suggest that Dox treatment increases the expression of inducible nitric oxide synthase (iNOS) and alters protein expression of endothelial nitric oxide synthase (eNOS) [6,7]. Dox-induced cardiac cell death and ROS production were attenuated by inhibition of NOS3 (eNOS) [9]. Dox also regulates many inflammatory genes to control downstream production of cytokines such as tumor necrosis factor-α (TNF-α), interleukin-1β (IL-1β) and interleukin-6 (IL-6) [6,7]. Toll-like receptors (TLRs) have also been shown to be involved in Dox-induced cardiomyopathy (DIC) where overexpression of TLR2 by Dox treatment worsens the cardiac function [14].

Oxidative and nitrosative stresses are also considered to be involved in the unfolded proteins response in the cardiac endoplasmic reticulum (ER), which activates several complex signaling pathways resulting in an ER stress. Unfolded proteins accumulate in ER under stress conditions. As a result, transmembrane protein sensors such as immunoglobulin-binding protein (Bip), also known as glucose-regulated protein 78 disassociates from DsRNA-activated protein kinase-like ER kinase (PERK), inositol-requiring kinase 1α (IRE1α) and activating transcription factor 6 (ATF6), as an adaptive response to maintain cellular homeostasis [15,16]. Bip is one of the most important chaperones in UPR regulation [17] which triggers cellular apoptotic pathways to remove cells under ER stress [18,19]. Recently, we have shown that Dox promoted UPR-related apoptotic protein CCAAT/enhancer-binding homologous protein (CHOP)/DNA damage-inducible transcript 3 (DDIT3) in normal as well as in hearts from tumor-bearing animals which was dependent on Bax—a mitochondrial proapoptotic protein [20].

Both iNOS-dependent and -independent pathways are mediated by OS-induced ER stress [21]. A potential role for ER stress in Dox-induced cardiotoxicity has been demonstrated by us and others [20,22]. Dox increases proinflammatory cytokines [6,7], which may have an important role in UPR signaling, possibly via iNOS activation and increased NO generation. It is possible that in unphysiologically higher levels, NO results in Bax-mediated UPR-related proapoptotic activation which ultimately leads to cardiac cell death in Dox-treated animals. Thus, we investigated whether the proapoptotic UPR signals in the development of DIC are regulated by iNOS/NO-induced inflammatory and adaptive responses.

## 2. Materials and Methods

### 2.1. Study Animals for In Vivo Dox Treatment

Approximately 8-week-old adult male Wistar rats (n = 17) were housed in a temperature-regulated room (22–24 °C) on a 12:12 h light–dark cycle. All experiments were conducted according to the guidelines of the Institutional Animal Care and Use Committee (protocol # 0032/2014) of the Universidade Nove de Julho, Sao Paulo, Brazil. Rats were given regular chow and water ad libitum. These animals (n = 11) were given 6 equal doses (2.5 mg/kg body weight intraperitoneal, i.p.) of Dox twice a week for a cumulative dose of 15 mg/kg over 3 weeks [7]. An equal volume of physiological saline was given to Control rats (n = 6). Two weeks after the last Dox injection, hearts from these rats were used for further studies.

### 2.2. Cardiomyocyte Isolation and Treatments

For in vitro cardiomyocyte isolation, male Sprague Dawley rats were used according to University of Manitoba Animal Care Committee approval and following the guidelines established by the Canadian Council on Animal Care (U of M protocol #19-058). As described earlier, viable cardiomyocytes were isolated from normal adult rat hearts (200–250 g) using a retrograde Langendorff perfusion method [23,24]. Cells were maintained in calcium-free M199 medium supplemented with 0.05% fetal bovine serum (FBS) at 5% CO_2_ in 37 °C incubator for 16 h. These cardiomyocytes were exposed to Dox (10 µM) for 24 h [6] in the presence (4 h pre-treatment) or absence of 100 µM of NO donor, S-Nitroso-N-acetyl-d,l-penicillamine (SNAP) [25] and/or 50 µM of NOS inhibitor N(ω)-nitro-L-arginine methyl ester (L-NAME) [26]. Control cells without Dox were incubated for same time points.

### 2.3. NO Production Assay

A commercially available kit from Oxford Biomedical Research (catalog no. NB98, Oxford, MI, USA) following manufacturer’s instructions was used to measure NO levels in the sample before and after 24 h of L-NAME and/or SNAP treatment. Absorbance was recorded at 540 nm using a spectrophotometer (Dynex Technologies, Chantilly, VA, USA). Sample concentrations were extrapolated on standard curve.

### 2.4. Western Blot

SDS-PAGE (8–12%) resolving gel was used for Western blot. A total of 30 µg protein was separated and allowed to transfer onto polyvinylidene fluoride (PVDF) membrane for 90 min at 200 mA at 4 °C [24]. Non-specific sites were blocked using 5% fat-free skimmed milk for 1 h at room temperature (RT). PVDF membranes were probed with specific antibodies (Santa Cruz Biotechnology, Paso Robles, CA, USA or Cell Signaling, Beverly, MA, USA) for overnight (12 h) at 4 °C. Next day, horseradish peroxidase (HRP)-conjugated secondary antibodies were used for 1 h at RT. Finally, membranes were washed with Tris-buffer saline (TBS) containing 0.1% Tween 20 (T20) and 1% BSA and bands were visualized using enhanced chemiluminescence (ECL) kit.

### 2.5. Immunoprecipitation

A two-step immunoprecipitation method was used as described earlier [23,24]. For pre-clearing, cell lysates were incubated with 5 µL of normal horse serum and 30 µL of protein A/G-conjugated Sepharose beads for 1 h at 4 °C. For antibody coupling, pre-cleared cell lysates were separated from beads by centrifugation at 7000× *g* for 5 min at 4 °C. Supernatants were further incubated with 2.5 µL of NF-κB p105/50 antibody (Cell Signaling, Beverly, MA, USA, cat ##3035) and 100 µL of the protein A/G gel beads for overnight at 4 °C. Samples were allowed to centrifuge at 12,000× *g* for 15 min at 4 °C to separate antigen–antibody complexes. After washing with lysis buffer, complexes were later eluted in 30 µL of SDS-sample buffer. Five microliter aliquots of each sample were used to run 8% SDS-PAGE resolving gel followed by western blots using TLR2 (Abcam, Cambridge, UK, cat #ab213676) or iNOS (Abcam, Cambridge, UK, cat #ab3523) primary antibody. Appropriate HRP-conjugated secondary antibodies were used for 1 h at RT and developed by using ECL substrate kit as described before [24].

### 2.6. Dual Immunofluorescence

#### 2.6.1. Adherent Cells

As described earlier [20], cardiomyocytes were allowed to adhere on chambered slides and fixed in 4% paraformaldehyde (PFA). Slides were washed with phosphate-buffered saline-Tween 20 (PBS-T) twice and permeabilized with 0.1% sodium citrate and 0.05% triton-X100 for 10 min at RT followed by 0.1% sodium borohydride treatment for 5 min at RT to quench autofluorescence. Slides were washed three times with PBS-T and incubated with 3% normal horse serum (NHS) for 1 h. Specific primary antibodies were used in co-localization of DDIT3 (Abcam, Cambridge, UK, cat #ab233121) together with iNOS, using Alexa 488, and 594 conjugated secondary antibodies (Invitrogen Life Technologies, Carlsbad, CA, USA). DAPI mounting media was used to locate nuclei. Slides were examined for co-expression of DDIT3/iNOS in Dox treated cardiomyocytes in the presence or absence of SNAP or L-NAME using the microscopic (Carl Zeiss., LSM 5 PASCAL, Jena, Germany) analysis setup attached with Zeiss AxioCam HRM camera HAL 100 lamp and quantified by using AxioVision 4.8 software.

#### 2.6.2. Cardiac Tissue Sections

Approximately 5–6 µm paraffin-embedded heart tissue sections were cut and mounted on microscopic slides, de-paraffinized in xylene and hydrated through graded series of alcohol as described earlier [20]. Tissue sections were permeabilized in 0.05% triton-X100 for 15 min at RT. To block endogenous peroxidase, enzymatic retrieval buffer (10 mM Tris base, 1 mM EDTA, 10 mM sodium citrate and 0.05% Tween 20, pH 9.0) was used, followed by 15 min incubation in 0.3% H_2_O_2_ in TBS and washed with PBS-T. Non-specific Fc-binding sites were blocked using 3% NHS for 1 h, washed twice with PBS-T and incubated with Bip (Abcam, Cambridge, UK, cat #21685) or Tom 20 primary antibodies together with iNOS or TLR2 using fluorophore Alexa 488 and/or Alexa 594-conjugated secondary antibody (Invitrogen, Life Technologies, Carlsbad, CA, USA). After washing, DAPI mounting media was used to detect nuclei. Expression of these antibodies was analyzed and quantified by the microscopic analysis as described above.

### 2.7. Statistical Analysis

Mean ± SE was used to assess data obtained from 3–4 independent experiments run in duplicate or triplicates. We used GraphPad Prism version 5.1 statistical software (GraphPad, La Jolla, CA, USA). One way ANOVA was used followed by post hoc Tukey’s test to test hypothesis. *p* < 0.05 was set as the level of significance.

## 3. Results

### 3.1. Dox-Induced ER Stress Mediates Inflammation and Mitochondrial iNOS

**Figure 1:**
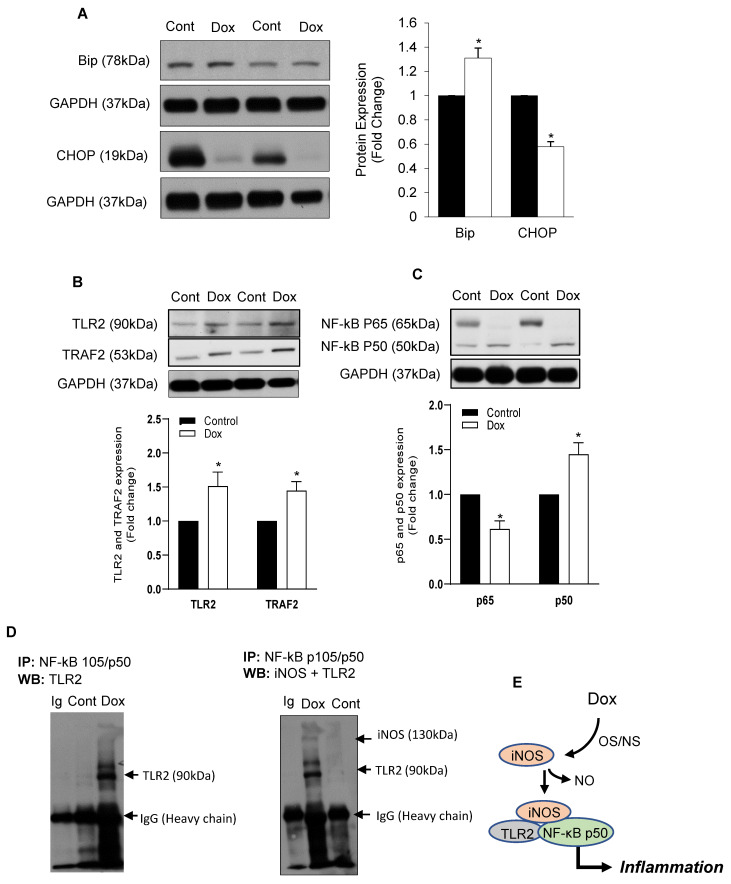
Dox promoted iNOS-mediated ER stress and Inflammation. Adult rat heart lysates from Dox-treated animals were probed for (**A**) Bip and CHOP, (**B**) TLR2 and TRAF2 and (**C**) NF-κBp65 (mouse Cell signaling #6956) and NF-κB p105/p50 (rabbit Cell signaling # 3035). GAPDH was used as a loading control. Histograms are Mean ± SE of 3 independent experiments done in duplicate. * *p* < 0.01 vs. respective controls. (**D**) Immunoprecipitation (IP) was done using NF-κB p105/p50 antibody followed by Western blot (WB) with specific TLR2 and iNOS antibodies. Immunoglobulin (Ig) was used as an input, negative control. The same blot was used for iNOS after TLR2 and stripping. (**E**) As immunoprecipitation with NF-κB p50 confirmed the presence of iNOS and TLR2 in the complex, a scheme of Dox promoted iNOS and activated TLR2 mediated inflammation is shown. OS/NS: oxidative/nitrosative stress. NO: Nitric oxide.

**Figure 2:**
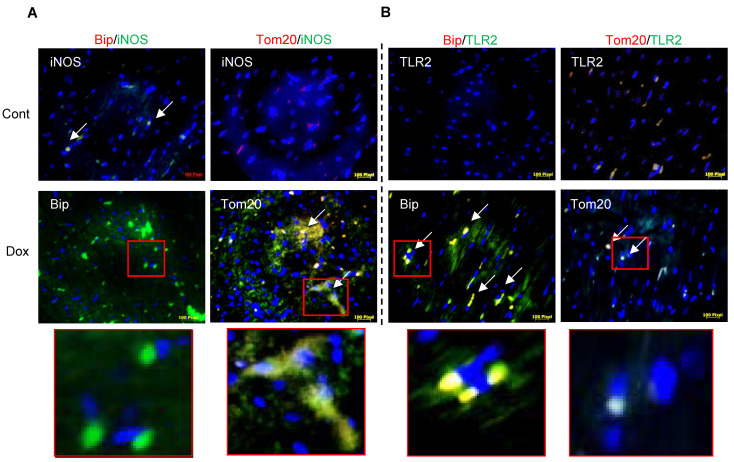
iNOS-regulated inflammation in ER stress and apoptosis in Dox-treated animals. Sections from Dox-treated and untreated hearts were processed for dual immunofluorescence staining for a co-localization of green fluorescence for iNOS (**A**) and TLR2 (**B**) together with red fluorescence for either Bip or Tom20. Areas in the red squares have been enlarged further. Heart sections were incubated with specific primary antibodies followed by secondary antibody labeled with Alexa 488 (Green) or Alexa 594 (Red), respectively. DA (blue) PI was used for nuclear staining. Scale bars are 100 µm.

We have recently reported that Dox-induced ER stress in cardiomyocytes is a delayed response of the Bax-mediated cell death process [20]. We now show that this delayed ER stress-mediated cell death pathway may be determined by iNOS-regulated inflammation during DIC. It has also been shown that Dox increased iNOS expression and NO levels while eNOS levels in the heart are decreased [7]. In the present study on rat heart lysates from Dox-treated rats, we noticed an increase in Bip with a decrease in CHOP activation in the heart (Figure 1A). Dox-induced increase in NO levels was also seen in isolated cardiomyocytes (Supplementary Figure S2A). Protein levels of inflammatory response markers TLR2 and TRAF2 were found to be increased by Dox in the heart lysates (Figure 1B). Dox increased transcription of NF-κB p50 as well as NF-κB p105 (Supplementary Figure S1) but not NF-κB p65 (Figure 1C). Immunoprecipitation experiments revealed that Dox increased binding of NF-κBp50 with TLR2 as well as with iNOS (Figure 1D). These data reflected that Dox induces iNOS activity and promotes downstream inflammatory processes via TLR2 and downstream NF-κBp50 (Figure 1E). Dual immunofluorescence staining for Bip and Tom20 co-localized with iNOS and TLR2, respectively, also confirmed that Dox-induced increase in iNOS was apparent with mitochondrial Tom20 but not with Bip expression (Figure 2A). Furthermore, Dox-induced increase in TLR2 expression was noted with Bip but not with Tom20 (Figure 2A compared with Figure 2B).

### 3.2. iNOS Synergistically Regulates Dox-ER Stress and Inflammatory Responses

**Figure 3:**
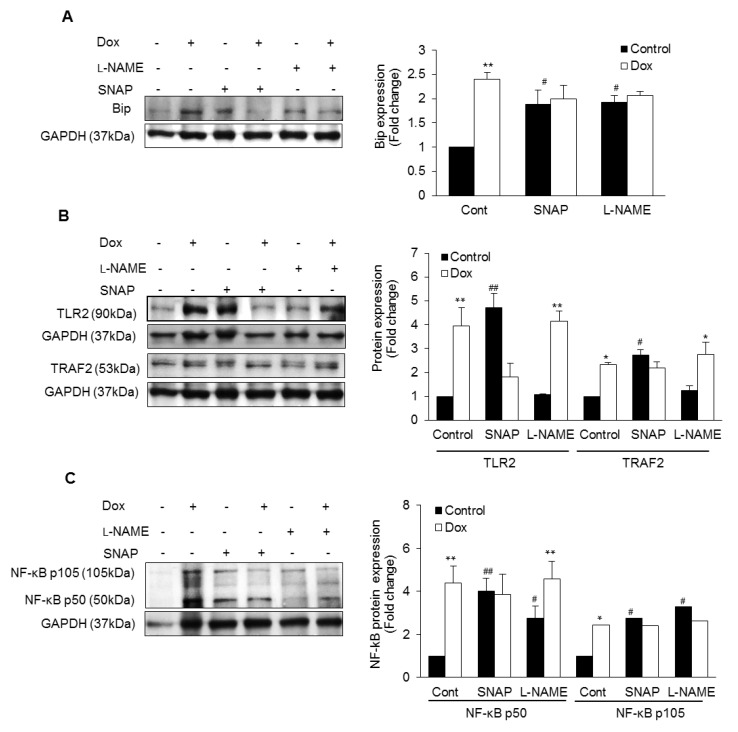
NO-mediated ER stress and inflammatory responses: Lysates from NO donor SNAP or NOS inhibitor L-NAME pre-incubated cardiomyocytes with or without doxorubicin (Dox) were immunoblotted for Bip (**A**), TLR2, TRAF2 (**B**) and NF-κB p105/p50 (**C**). GAPDH was used as loading control. Histograms are Mean ± SE of 3 independent experiments done in duplicate. * *p* < 0.05 or ** *p* < 0.002 vs. respective control and # *p* < 0.05 or ## *p* < 0.002 vs. Control.

Dox-treated isolated cardiomyocytes were analyzed for ER stress-induced inflammatory responses. iNOS is a major source of NO production as inhibition of NOS with L-NAME reduced the production of NO (Supplementary Figure S2A). Dox significantly increased NO production in control cardiomyocytes as well as in the presence of L-NAME (Supplementary Figure S2A). Dox-induced increase in NO was not seen in the presence of SNAP (Supplementary Figure S2A). Dox significantly increased Bip (Figure 3A) and DDIT3 expression (Supplementary Figure S2B). Increased Bip by Dox was inhibited in presence of NO donor (SNAP) or NOS inhibitor (L-NAME) compared to Dox alone (Figure 3A). Dox promoted TLR2 as well as TRAF2 (Figure 3B). Nevertheless, Dox maintained this increase in TLR2 and TRAF2 expression in inhibition of NOS, while levels for both were decreased in presence of NO donor (Figure 3B). Downstream transcription factor NF-kB p50 was significantly higher in presence of Dox. NO donor also caused a significant increase in NF-κB p50. However, Dox did not cause any further increase (Figure 3C). Inhibition of NOS also increased NF-kB p50 activity and Dox caused a further increase (Figure 3C). Both NO donor and NOS inhibition caused an increase in NF-κB p105 levels, while Dox had no further effect (Figure 3C). Dox increased SERCA-2a expression in control cardiomyocytes which was promoted by NO donor and blunted by NOS-inhibition (Supplementary Figure S2C).

### 3.3. iNOS Regulates ER Stress-Induced Apoptosis

**Figure 4:**
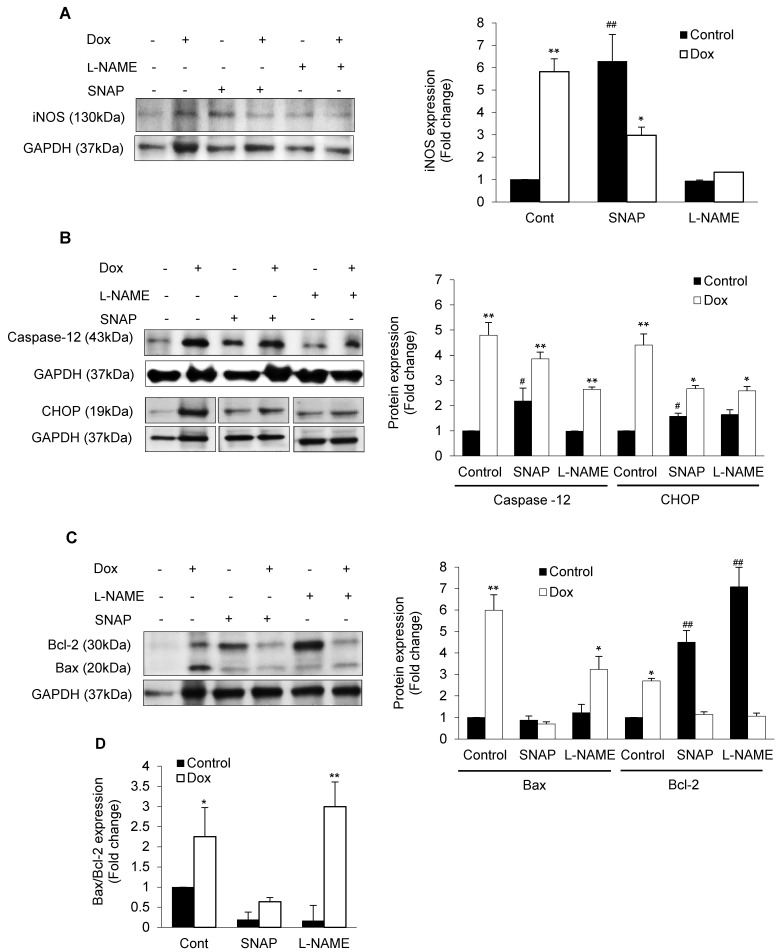
NO-mediated ER stress-induced apoptosis: Isolated cardiomyocytes were incubated with or without Dox following pre-incubation with NO donor SNAP or iNOS inhibitor L-NAME and were analyzed for iNOS (**A**), Caspase 12 and CHOP or DDIT3 (**B**), and Bax and Bcl-2 (**C**). GAPDH was used as loading control. (**D**) Histograms are also shown for western blot quantitative fold change of Bax/Bcl2 ratio. These histograms are mean ± SE of 3 independent experiments done in duplicate. * *p* < 0.05 or ** *p* < 0.002 vs. respective control and # *p* < 0.05 or ## *p* < 0.002 vs. Control.

**Figure 5:**
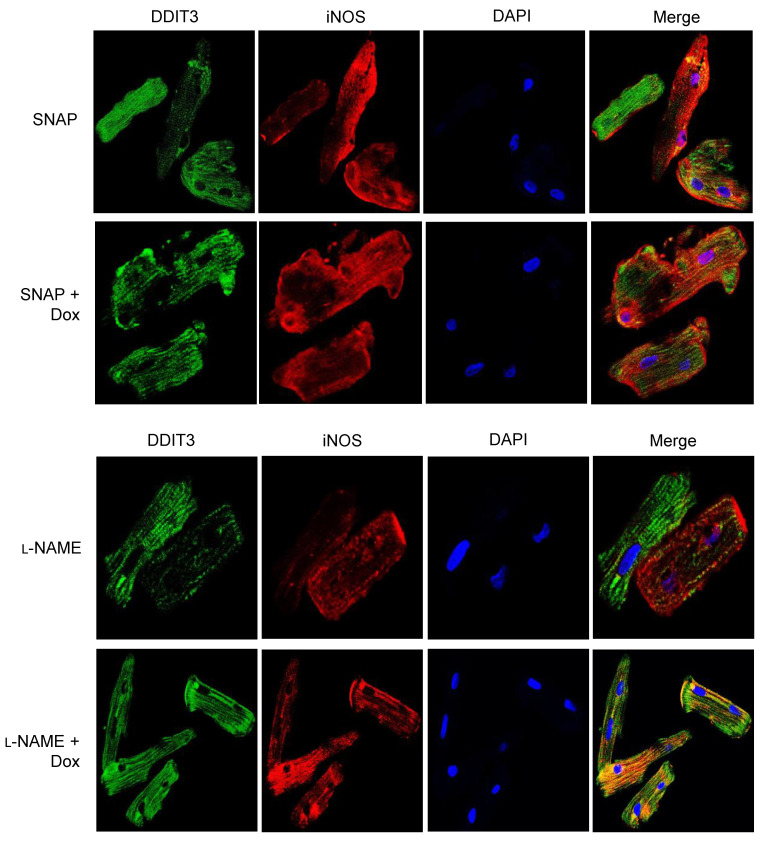
iNOS-dependent ER stress-induced apoptosis by Dox: Isolated cardiomyocytes pre-incubated with NO donor (SNAP) or NOS inhibitor (L-NAME) following treatment with or without doxorubicin (Dox) were fixed in 4% paraformaldehyde (PFA). For co-localization of DDIT3 and iNOS study, fixed cardiomyocytes were incubated with specific antibodies followed by secondary antibody labeled with Alexa 488 (Green) or Alexa 594 (Red) respectively. DAPI was used for nuclear staining. Each panel is image enlarged from a picture taken at 60× in oil.

As suggested earlier, Dox-induced iNOS expression in isolated cardiomyocytes and NO supplementation with SNAP increased iNOS expression, which was inhibited by Dox (Figure 4A). Interestingly, there was an increase in CHOP activation in Dox-treated cardiomyocytes in presence or absence of NO compared to control (Figure 4B). Similarly, Dox induced DDIT3 in either presence of NO or inhibition of NOS (Supplementary Figure S2B). Increase in DDIT3/CHOP may have been stimulated by excess of NO in the system. Likewise, Caspase 12 was found to be increased by Dox in presence of SNAP or L-NAME (Figure 4B). In contrast, increased mitoBax by Dox was inhibited by NO supplementation or by NOS inhibition (Figure 4C). As expected, both NO and inhibition of iNOS increased Bcl-2 and reduced Dox-induced Bax activation (Figure 4C). While we compared the Bax/Bcl2 ratio, it seems that Dox was promoted 3-fold in the ratio in NOS inhibition (Figure 4D). In consonant with the western blot data, our co-localization study revealed that Dox promoted DDIT3 as well as iNOS activation (Supplementary Figure S3) in presence of NO and L-NAME, suggesting an ER stress-induced apoptosis regulated by iNOS (Figure 5).

## 4. Discussion

NO-induced apoptosis is mediated by mitochondrial damage [27,28], however the cell death cascade caused by NO/iNOS during Dox-induced cardiotoxicity has not been fully explained. It is also reported that ER stress signaling is important for NO-induced apoptosis which is regulated through CHOP/GADD153 [28,29,30,31]. Excess NO disturbs Ca^2+^-dependent protein folding processes in ER [32]. NO activates p53 and PPAR which is mediated by a mitochondrial pathway [33]. Data also suggest that cells treated with NO reduced ER Ca^2+^ concentrations and induces ATF6/CHOP mediated apoptosis even in absence of the p53 gene [29,30]. Therefore, NO produced from iNOS can be an activator of ER stress pathways controlled by mitochondrial p53 independently. Dox-induced adaptive responses attenuate ER stress in cancer cells [20]; however, its delayed effect in cardiomyocyte ER stress is not completely understood.

It has been reported that increased UPR chaperone Bip (GRP78) is an adaptive response to Dox treatment by which carboxyl terminal Lys-Asp-Glu-Leu (KDEL) sequence of Grp78 binds to its receptor and returns Bip to the ER [34]. Dox increased Bip suggests an activation of the receptor for transporting the protein–receptor complex back to the ER. Our recent study showed that Dox activated Bax at the initial hours (3 h), while Bip and DDIT3 were noted at later time points in cardiomyocytes [20]. Such delayed DDIT3 activation together with Bax suggests a mitochondrial-mediated Bax activation may have triggered ER stress-mediated apoptosis. We know that certain calcium (Ca^2+^) channel receptors such as sarcoendoplasmic reticulum Ca^2+^-ATPases (SERCAs) determine Ca^2+^ movements across the ER membrane [35]. We saw an increase in SERCA-2a by Dox which was promoted by NO donor and mitigated by NOS inhibition (Supplementary Figure S2C). Thus, Dox treatment may have resulted in the depletion of ER calcium and its overload in the cytoplasm triggering apoptosis via activated DDIT3/CHOP and ER-resident caspase-12 following mitochondrial dysfunction.

It is well known that iNOS is a key target gene for NF-κB activation and capable of regulating its activity by inhibiting or enhancing downstream pathways [36]. Extensive nitrosylation of a member protein in Dox-treated animals increases activities of the transcription factors p53 as well as rel A/p65 subunit of NF-κB in Dox-induced inflammation [6,7]. Increased NF-kB activity is associated with increased levels of proinflammatory IL-1β and TNF-α cytokines which may have further induced production of NO via activation of iNOS [7]. Nevertheless, NF-κB is one of the important pathways activated by TRAF2 and TRAF5 [37] leading to ER stress [38]. It is reported that UPR triggers cell death by promoting intrinsic apoptotic pathway through TRAF2. TRAF2 interacts with apoptosis signaling kinase 1 (Ask1) and phosphorylate the c-Jun N-terminal kinase (JNK) [39]. Increased TRAF2 by Dox suggested that TRAF2 may have activated NF-κB p50-mediated TLR2 response. TLR2 together with NF-κB p50 activation of NF-κB is an important component of a positive feedforward response where iNOS acted as a connecting driver of inflammatory response leading to cardiotoxicity [40]. It is also reported that SNAP blocks cytokine-induced increase in iNOS activity, nuclear translocation of cytosolic NF-κB p65 subunit and inhibitory kappa B (IκB) kinase (IKK) degradation [36,41,42,43]. Similar to these findings, SNAP blocks Dox-induced iNOS, NF-κB p105/p50 and downstream TLR2 mediated inflammation. NO donor increases iNOS activity in a positive feedback process and Dox inhibits NO-induced iNOS activity with no change in eNOS activity (Supplementary Figure S2B). Interestingly, we also noticed that Dox increased eNOS in the absence of NO or NOS suggesting that Dox independently enhance eNOS and NO in order to maintain an adaptive response. On the other hand, Dox also reduced NO-induced Bip, TLR2, TRAF2, NF-κB p105 and Bax, whereas it increased the expression of SERCA 2a, Caspase 12, CHOP, suggesting that Dox engaged ER-mediated apoptosis in DIC. Furthermore, inhibition of NOS promoted Dox-induced TLR2 and TRAF2 responses thus suggesting that Dox independently induces NO which may have acted as a modulator of Dox-induced ER-stress and inflammation as addition of NO donor, SNAP decreases such inflammatory reactions via reducing NF-κB p105 transcription factor. Dox induced CHOP as well as Caspase 12 activity in either presence of NO or inhibition of NOS. Increased Bcl-2 response by direct supplementation of NO or inhibition of NOS may have suppressed Dox-induced Bax expression. On the other hand, Dox in the presence of NO promoted the CHOP and DDIT3 expression without change in iNOS suggesting that Dox-induced DDIT3/CHOP is NO independent however iNOS dependent.

Inflammatory response and apoptosis are thought to be happening simultaneously during DIC. We have reported that TLR2 dominates over TLR4 regulation through NF-kB mediated pathway and activation of IRAK2/M [23,24]. Later, we have shown that increased OxPCs may have worsen the I/R injury via TLR2 upregulation controlled by LOX-1 [44]. We now assume that iNOS is a major downstream mediator of inflammation in cardiomyocytes and causes DIC. We and others have previously shown that iNOS is a critical player to cause DIC [6,7,45]. DIC may also be characterized by an aberrant UPR signaling leading to the suppression of many adaptive responses [22] via increased NO and iNOS. It is proposed that a decrease in cardiomyocyte defense mechanisms against ER stress contributes to Dox susceptibility in hearts from tumor-bearing animals [20] and is suggested to be controlled by iNOS-regulated TLR2 activity. DDIT3 or CHOP expression in cardiomyocyte is mitochondrial Bax-dependent and promoted by iNOS to induce downstream inflammatory signals by TLR2. Our data reflect that paradoxically NO may also be required for cell survival as an early adaptive response via Bcl-2 which may be promoted by iNOS in order to achieve protection against DIC. With regard to downstream Dox-mediated UPR signaling, DDIT3/CHOP was markedly induced in cardiomyocytes with increased NO production which may have later promoted inflammatory signaling via TLR2. It is apparent that Dox retained the ability to induce ER stress-induced apoptosis even after inhibition of NO formation where Caspase 12 may have involved in an activation of downstream DDIT3/CHOP. Moreover, inhibition of NOS by L-NAME is associated with the amelioration of inflammatory responses as there was a reduced TLR2 expression. These data indicate that iNOS/NO are required to promote inflammation following ER stress-induced apoptosis.

### Study Limitations

Despite our comprehensive approach on the roles of ER stress, iNOS/NO and inflammation in the mediation of Dox-induced cardiomyopathy, our study does have some limitations. A proteomic analysis of nitrosylated components by Dox would shed light on more precise protein changes. Similarly, a focused analysis of endothelial (first sensor of circulating Dox) effects could be useful. NO donor (SNAP) and/or NOS inhibitor (L-NAME) studies were done only in isolated myocytes and not in the whole animals. Heart, being an organ with a very limited regenerative capacity, faces increased consequences of Dox-induced cardiomyocyte loss. In the interest of keeping a focus in our study, we did not get into these aspects of the analysis and hopefully did deliver a message on the subcellular basis of the role of ER stress in doxorubicin-induced cardiomyopathy.

## 5. Conclusions

**Figure 6:**
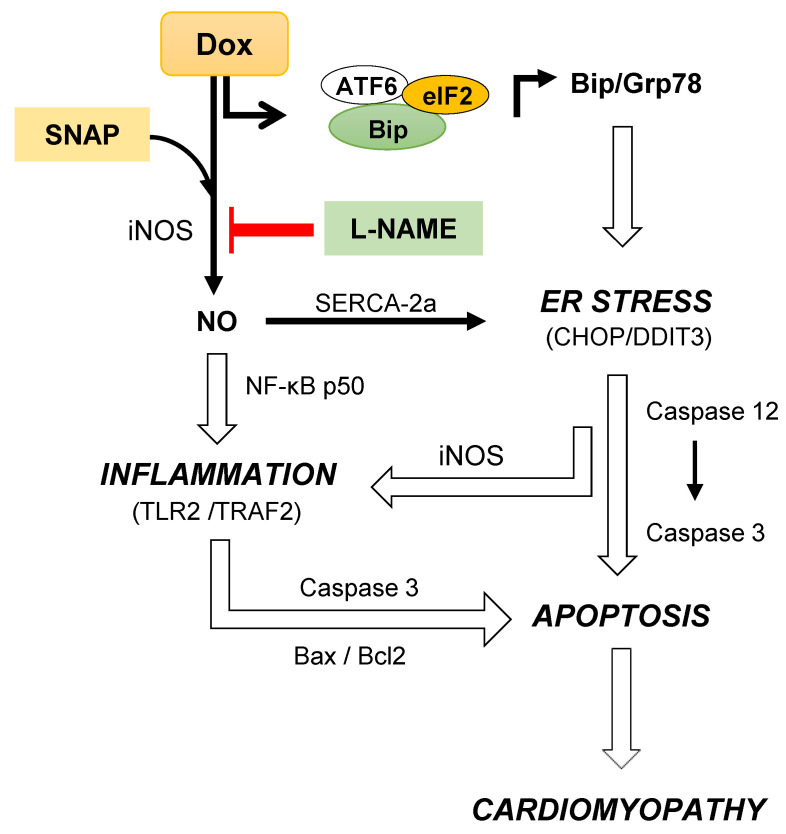
Dox -induced synergistic inflammatory and ER stress responses leading to apoptosis and cardiomyopathy: Dox dissociates Bip from the Bip-ATF6-eIF2α complex suggested to cause an ER stress and increase in UPR signaling [20]. Such an adaptive response induces iNOS and TLR2 activation following through the regulation of NF-κB p50 and as a result enhancing inflammation. Dox-induced iNOS activation also increases NO and triggers nitrosative stress in isolated cardiomyocytes and in the heart [6,7]. As reported earlier, it is evident that NO donor increases iNOS activity and Dox paradoxically inhibited NO-induced iNOS without change in eNOS activity. Increased iNOS-mediated TLR2 activation results in an ER-induced apoptosis and cardiomyopathy via CHOP/DDIT3 and intrinsic Caspase 3 activation. NOS inhibitor L-NAME may mitigate such Dox-induced ER stress-mediated inflammation, apoptosis and ultimately cardiomyopathy.

Very recently, we have shown that a dual mode of Dox action in cardiomyocytes and in isolated tumors is dependent on an interplay between proapoptotic protein Bax and UPR-related DDIT3 apoptotic protein through AFT6/Bip signaling [20]. In continuation, here we demonstrate that iNOS-induced NO promoted CHOP/DDIT3 as well as activation of caspases 12 and 3 suggesting an ER stress-induced apoptosis, regulated by iNOS (Figure 6). Doxorubicin-induced ER stress promoted inflammation via the mitochondrial iNOS and Bax activation. Mitigation of iNOS/NO may reverse both ER and mitochondrial stresses in achieving prevention against DIC (Figure 6).

## Data Availability

Data are contained within the article and supplementary material.

## Supplementary Materials

The following are available online at https://www.mdpi.com/article/10.3390/antiox10121897/s1, Figure S1: Dox treated and untreated heart cell lysates were run for western blot for NF-κB p105/50, Figure S2: Dox increased NO levels and DDIT3 in presence of iNOS inhibitor, Figure S3: Co-localization of DDIT3 and iNOS in isolated cardiomyocytes

## References

[1] Lefrak E.A., Pitha J., Rosenheim S., Gottlieb J.A. (1973). A clinicopathologic analysis of adriamycin cardiotoxicity. Cancer.

[2] Singal P.K., Iliskovic N. (1998). Doxorubicin-induced cardiomyopathy. N. Engl. J. Med..

[3] Songbo M., Lang H., Xinyong C., Bin X., Ping Z., Liang S. (2019). Oxidative stress injury in doxorubicin-induced cardiotoxicity. Toxicol. Lett..

[4] Cappetta D., De Angelis A., Sapio L., Prezioso L., Illiano M., Quaini F., Rossi F., Berrino L., Naviglio S., Urbanek K. (2017). Oxidative Stress and Cellular Response to Doxorubicin: A Common Factor in the Complex Milieu of Anthracycline Cardiotoxicity. Oxid. Med. Cell Longev..

[5] Ludke A.R., Al-Shudiefat A.A., Dhingra S., Jassal D.S., Singal P.K. (2009). A concise description of cardioprotective strategies in doxorubicin-induced cardiotoxicity. Can. J. Physiol. Pharm..

[6] Akolkar G., Bagchi A.K., Ayyappan P., Jassal D.S., Singal P.K. (2017). Doxorubicin-induced nitrosative stress is mitigated by vitamin C via the modulation of nitric oxide synthases. Am. J. Physiol. Cell Physiol..

[7] Akolkar G., da Silva Dias D., Ayyappan P., Bagchi A.K., Jassal D.S., Salemi V.M.C., Irigoyen M.C., De Angelis K., Singal P.K. (2017). Vitamin C mitigates oxidative/nitrosative stress and inflammation in doxorubicin-induced cardiomyopathy. Am. J. Physiol. Heart Circ. Physiol..

[8] Mukhopadhyay P., Rajesh M., Bátkai S., Kashiwaya Y., Haskó G., Liaudet L., Szabó C., Pacher P. (2009). Role of superoxide, nitric oxide, and peroxynitrite in doxorubicin-induced cell death in vivo and in vitro. Am. J. Physiol. Heart Circ. Physiol..

[9] Neilan T.G., Blake S.L., Ichinose F., Raher M.J., Buys E.S., Jassal D.S., Furutani E., Perez-Sanz T.M., Graveline A., Janssens S.P. (2007). Disruption of nitric oxide synthase 3 protects against the cardiac injury, dysfunction, and mortality induced by doxorubicin. Circulation.

[10] Farías J.G., Molina V.M., Carrasco R.A., Zepeda A.B., Figueroa E., Letelier P., Castillo R.L. (2017). Antioxidant Therapeutic Strategies for Cardiovascular Conditions Associated with Oxidative Stress. Nutrients.

[11] Goncalves L.T., Erthal F., Corte C.L., Muller L.G., Piovezan C.M., Nogueira C.W., Rocha J.B. (2005). Involvement of oxidative stress in the pre-malignant and malignant states of cervical cancer in women. Clin. Biochem..

[12] Pizarro M., Troncoso R., Martínez G.J., Chiong M., Castro P.F., Lavandero S. (2016). Basal autophagy protects cardiomyocytes from doxorubicin-induced toxicity. Toxicology.

[13] Singal K.P., Li T., Kumar D., Danelisen I., Iliskovic N. (2000). Adriamycin-induced heart failure: Mechanism and modulation. Mol. Cell. Biochem..

[14] Ma Y., Zhang X., Bao H. (2012). Toll-like receptor (TLR) 2 and TLR4 differentially regulate doxorubicin induced cardiomyopathy in mice. PLoS ONE.

[15] Lee A.S. (2005). The ER chaperone and signaling regulator GRP78/BiP as a monitor of endoplasmic reticulum stress. Methods.

[16] Bertolotti A., Zhang Y., Hendershot L.M., Harding H.P., Ron D. (2000). Dynamic interaction of BiP and ER stress transducers in the unfolded-protein response. Nat. Cell Biol..

[17] Hendershot L.M. (2004). The ER function BiP is a master regulator of ER function. Mt. Sinai J. Med..

[18] Kaufman R.J. (2002). Orchestrating the unfolded protein response in health and disease. J. Clin. Investig..

[19] Urano F., Wang X., Bertolotti A., Zhang Y., Chung P., Harding H.P., Ron D. (2000). Coupling of stress in the ER to activation of JNK protein kinases by transmembrane protein kinase IRE1. Science.

[20] Bagchi A.K., Malik A., Akolkar G., Zimmer A., Belló-Klein A., De Angelis K., Jassal D.S., Fini M.A., Stenmark K.R., Singal P.K. (2021). Study of ER stress and apoptotic proteins in the heart and tumor exposed to doxorubicin. Biochim. Biophys. Acta Mol. Cell Res..

[21] Hsieh Y.H., Su I.J., Lei H.Y., Lai M.D., Chang W.W., Huang W. (2007). Differential endoplasmic reticulum stress signaling pathways mediated by iNOS. Biochem. Biophys. Res. Commun..

[22] Yarmohammadi F., Rezaee R., Haye A.W., Karimi G. (2021). Endoplasmic reticulum stress in doxorubicin-induced cardiotoxicity may be therapeutically targeted by natural and chemical compounds: A review. Pharmacol. Res..

[23] Bagchi A.K., Akolkar G., Mandal S., Ayyappan P., Yang X., Singal P.K. (2017). Toll-like receptor 2 dominance over Toll-like receptor 4 in stressful conditions for its detrimental role in the heart. Am. J. Physiol. Heart Circ. Physiol..

[24] Bagchi A.K., Sharma A., Dhingra S., Lehenbauer Ludke A.R., Al-Shudiefat A.A., Singal P.K. (2013). Interleukin-10 activates Toll-like receptor 4 and requires MyD88 for cardiomyocyte survival. Cytokine.

[25] Pravdic D., Vladic N., Cavar I., Bosnjak Z.J. (2012). Effect of nitric oxide donors S-nitroso-N-acetyl-DL-penicillamine, spermine NONOate and propylamine propylamine NONOate on intracellular pH in cardiomyocytes. Clin. Exp. Pharm. Physiol..

[26] Strijdom H., Muller C., Lochner A. (2004). Direct intracellular nitric oxide detection in isolated adult cardiomyocytes: Flow cytometric analysis using the fluorescent probe, diaminofluorescein. J. Mol. Cell Cardiol..

[27] Brown G.C. (2007). Nitric oxide and mitochondria. Front. Biosci..

[28] Xu W., Liu L., Charles I.G., Moncada S. (2004). Nitric oxide induces coupling of mitochondrial signalling with the endoplasmic reticulum stress response. Nat. Cell Biol..

[29] Kawahara K., Oyadomari S., Gotoh T., Kohsaka S., Nakayama H., Mori M. (2001). Induction of CHOP and apoptosis by nitric oxide in p53-deficient microglial cells. FEBS Lett..

[30] Gotoh T., Oyadomari S., Mori K., Mori M. (2002). Nitric oxide-induced apoptosis in RAW 264.7 macrophages is mediated by endoplasmic reticulum stress pathway involving ATF6 and CHOP. J. Biol. Chem..

[31] Gotoh T., Mori M. (2006). Nitric oxide and endoplasmic reticulum stress. Arter. Thromb. Vasc. Biol..

[32] Oyadomari S., Araki E., Mori M. (2002). Endoplasmic reticulum stress-mediated apoptosis in pancreatic beta-cells. Apoptosis.

[33] Messmer U.K., Brüne B. (1996). Nitric oxide-induced apoptosis: p53-dependent and p53-independent signalling pathways. Biochem. J..

[34] Yamamoto K., Fujii R., Toyofuku Y., Saito T., Koseki H., Hsu V.W., Aoe T. (2001). The KDEL receptor mediates a retrieval mechanism that contributes to quality control at the endoplasmic reticulum. EMBO J..

[35] East J.M. (2000). Sarco(endo)plasmic reticulum calcium pumps: Recent advances in our understanding of structure/function and biology (review). Mol. Membr. Biol.

[36] Katsuyama K., Shichiri M., Marumo F., Hirata Y. (1998). NO inhibits cytokine-induced iNOS expression and NF-kappaB activation by interfering with phosphorylation and degradation of IkappaB-alpha. Arter. Thromb. Vasc. Biol..

[37] Tada K., Okazaki T., Sakon S., Kobarai T., Kurosawa K., Yamaoka S., Hashimoto H., Mak T.W., Yagita H., Okumura K. (2001). Critical roles of TRAF2 and TRAF5 in tumor necrosis factor-induced NF-kappa B activation and protection from cell death. J. Biol. Chem..

[38] Zhang K., Shen X., Wu J., Sakaki K., Saunders T., Rutkowski D.T., Back S.H., Kaufman R.J. (2006). Endoplasmic reticulum stress activates cleavage of CREBH to induce a systemic inflammatory response. Cell.

[39] Kato H., Nakajima S., Saito Y., Takahashi S., Katoh R., Kitamura M. (2012). mTORC1 serves ER stress-triggered apoptosis via selective activation of the IRE1-JNK pathway. Cell Death Differ..

[40] Mancilla T.R., Iskra B., Aune G.J. (2019). Doxorubicin-Induced Cardiomyopathy in Children. Compr. Physiol..

[41] Taylor B.S., Kim Y.M., Wang Q., Shapiro R.A., Billiar T.R., Geller D.A. (1997). Nitric oxide down-regulates hepatocyte-inducible nitric oxide synthase gene expression. Arch. Surg..

[42] Prabhu S.D. (2004). Cytokine-induced modulation of cardiac function. Circ. Res..

[43] Marshall H.E., Stamler J.S. (2001). Inhibition of NF-kappa B by S-nitrosylation. Biochemistry.

[44] Bagchi A.K., Surendran A., Malik A., Jassal D.S., Ravandi A., Singal P.K. (2020). IL-10 attenuates OxPCs-mediated lipid metabolic responses in ischemia reperfusion injury. Sci. Rep..

[45] Cole M.P., Chaiswing L., Oberley T.D., Edelmann S.E., Piascik M.T., Lin S.M., Kiningham K.K., St Clair D.K. (2006). The protective roles of nitric oxide and superoxide dismutase in adriamycin-induced cardiotoxicity. Cardiovasc. Res..

