# Supplementary material for: Endoplasmic Reticulum Stress Promotes iNOS/NO and Influences Inflammation in the Development of Doxorubicin-Induced Cardiomyopathy
Source: Antioxidants (Basel). 2021 Nov 26;10(12):1897. doi: 10.3390/antiox10121897 (PMC8750247; doi:10.3390/antiox10121897)
Supplement: Supplementary file 1 [file antioxidants-10-01897-s001.zip › antioxidants-1448983-supplementary.pdf]

Supplementary Materials

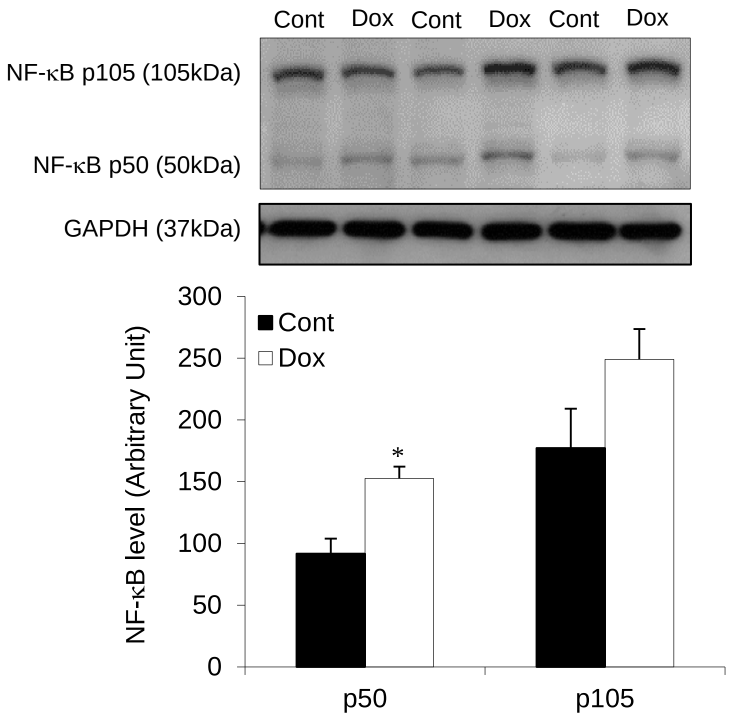

Supplementary Figure S 1: Dox treated and untreated heart cell lysates were run for western blot for NF-κB p105/50. GAPDH was used as an internal control. Histograms are Mean ± SE of three independent experiments done in duplicate. * $P < 0.05$ vs. control.

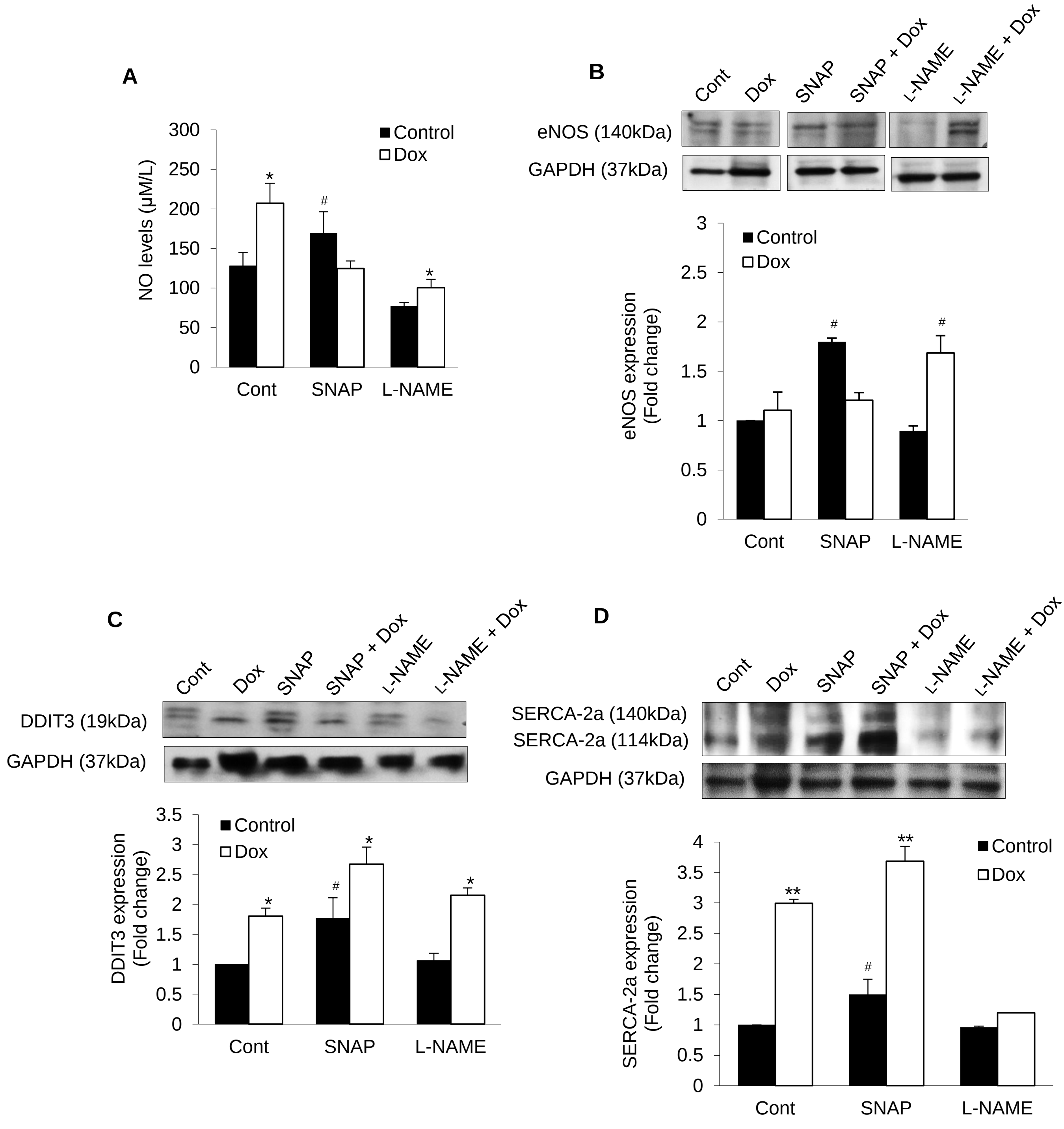

Supplementary Figure S 2: Dox increased NO levels and DDIT3 in presence of iNOS inhibitor. NO donor (SNAP) or NOS inhibitor (L-NAME) pre-incubated cardiomyocytes treated with or without doxorubicin (Dox) were used to measure NO levels using Griess reagent (**A**). Lysates were also run for western blot for eNOS (**B**) DDIT3 (**C**) and for SARCA 2a (**D**). GAPDH was used as an internal control (also shown in figure 4B). Histograms are Mean ± SE of three independent experiments done in duplicate. * P < 0.05 or ** P < 0.002 vs. respective controls; # P < 0.05 vs. control.

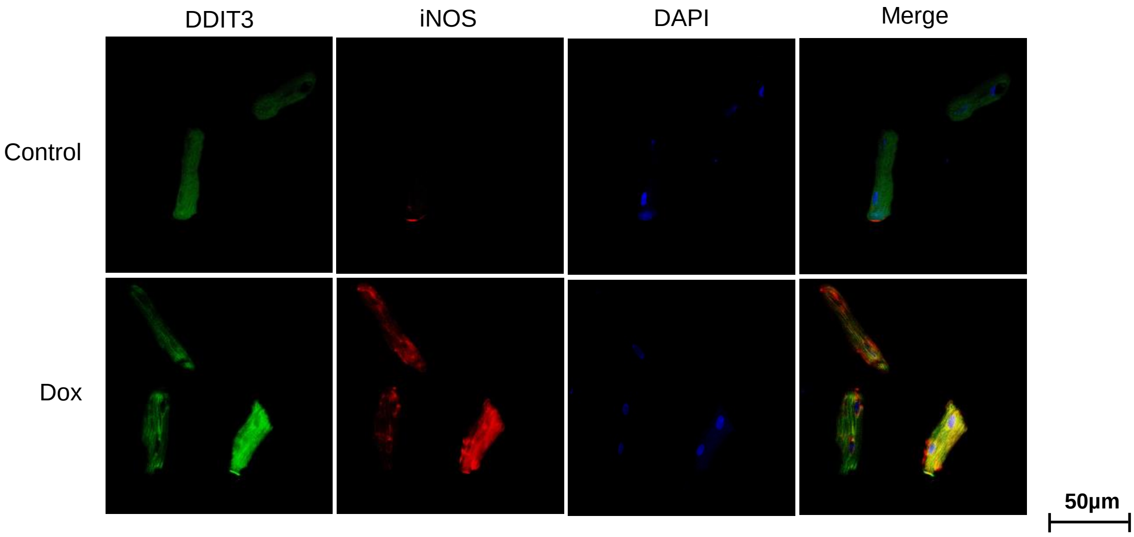

Supplementary Figure S 3: Co-localization of DDIT3 and iNOS in isolated cardiomyocytes. Samples from Dox treated cardiomyocytes were fixed in 4% paraformaldehyde (PFA) and incubated with specific antibodies for mouse DDIT3 and rabbit iNOS followed by secondary antibody labeled with Alexa 488 (Green) or Alexa 594 (Red) respectively. DAPI was used for nuclear staining. Scale bars is 50µm.
